# The Roles of Parasitoid Foraging for Hosts, Food and Mates in the Augmentative Control of Tephritidae

**DOI:** 10.3390/insects3030668

**Published:** 2012-07-20

**Authors:** John Sivinski, Martin Aluja

**Affiliations:** 1 USDA-ARS, Center for Medical, Agricultural and Veterinary Entomology, 1600 SW23rd Drive, Gainesville, FL 32604, USA; 2 Instituto de Ecología, A.C., Apartado Postal 63, Xalapa 91000, Veracruz, Mexico; E-Mail: martin.aluja@inecol.edu.mx

**Keywords:** Tephritidae, Braconidae, *Anastrepha*, *Ceratitis*, *Bactrocera*, Opiinae, Figitidae, signals, cues

## Abstract

Ultimately, the success of augmentative fruit fly biological control depends upon the survival, dispersal, attack rate and multi-generational persistence of mass-reared parasitoids in the field. Foraging for hosts, food and mates is fundamental to the above and, at an operational level, the choice of the parasitoid best suited to control a particular tephritid in a certain environment, release rate estimates and subsequent monitoring of effectiveness. In the following we review landscape-level and microhabitat foraging preferences, host/fruit ranges, orientation through environmental cues, host vulnerabilities/ovipositor structures, and inter and intraspecific competition. We also consider tephritid parasitoid mating systems and sexual signals, and suggest the directions of future research.

## 1. Introduction

Tephritid-parasitoid guilds worldwide include numerous pteromalid, eupelmid, chalcid and diapriid pupal parasitoids as well as eulophid, and figitid larval parasitoids. However, species of opiine braconids are the most diverse, abundant and agriculturally important, particularly in terms of augmentative release [1,2]. Ultimately, the successful deployment of these opiines (and occasionally other types of parasitoids) depends upon their survival, dispersal, attack rate and multi-generational persistence in the field. Foraging for hosts, food and mates is fundamental to all of these and, at an operational level, the choice of parasitoid(s) best suited to control a particular tephritid in a certain environment, release rate estimates, subsequent monitoring of effectiveness and the formulation of mass-rearing quality control standards.

In the following we review for what fruit fly parasitoids forage and the means they use to obtain these resources. The implications of foraging behaviors for augmentative biological control are then discussed as are directions for future research.

## 2. Foraging Behaviors

### 2.1. Foraging for Hosts

#### 2.1.1. Visual Cues

It is a truism that fruit are colorful. Tephritid parasitoids use vision while foraging for hosts [3] and color, as well as shape and size, could represent host-foraging cues. While some parasitoids have innate preferences for colors [4], others come to prefer particular colors after they become associated with host encounters [5]. Evidence for color responses in opiine braconids is somewhat mixed. *Diachasmimorpha longicaudata* (Ashmead) in field cages responded differently to fruit-baited traps on the basis of their color [6]. Males had a preference for yellow and green and, while females had less variance in their responses, red and blue were less attractive than yellow. However, it was unclear if they responded to the colors’ hues or reflectance, and in the laboratory, Leyva, Browning and Gilstrap [7] were unable to find any innate preference by *D. longicaudata* for orange, yellow, red or green.*Fopius arisanus* (Sonan) has also been the subject of several color-response investigations, which again have yielded somewhat ambiguous results. In the absence of fruit odors *F. arisanus* were less likely to land on yellow than black or green “targets” and Rousse, Chiroleu, Domberg, and Quilici [8] concluded there was no evidence for hue-discrimination but there was orientation towards darker objects that in nature might represent fruit against the foliage. However, under more controlled conditions females presented with fruit-odor baited targets of a variety of colors, shapes and sizes preferred the larger of dark-yellow spheres [9]. *Psyttalia concolor* (Szépligeti) had no innate preferences for color, shape or pattern, but did learn to associate colors with the presence of host larvae [10].

#### 2.1.2. Chemical Cues

**Figure 1:**
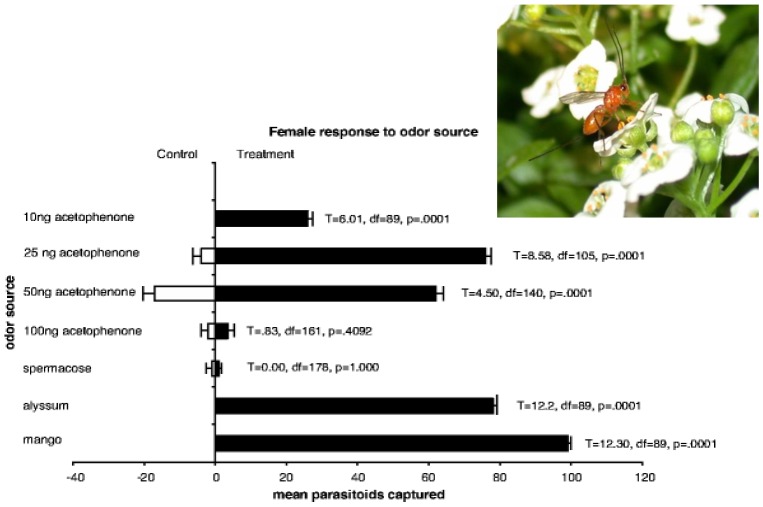
A synthetic attractant/trap to monitor the survival and dispersal of argumentatively released parasitoids would contribute to the management of area-wide control programs. Female *D. longicaudata* fly toward volatiles of the alyssum flower [*Lobularia maritima*, (Brassicaceae)] but not to those of *Spermacoce verticillata* (Rubiaceae). Acetophenone is a major component of the first, but is absent from the later, and acetophenone is itself attractive. Although female, but not male, *D. longicaudata* are attracted to the flowers (inset) there is little evidence they feed upon its nectar and the ultimate cause of the positive response remains a mystery (although the chemical homologue para-ethyl-acetophenone is in volatiles released by many tephritid larvae) [14].

Chemicals associated with fruit flies/fruit both attract (host-location cues) and arrest (host-habitat cues) fruit fly parasitoids [11]. For example, a preliminary list of the chemicals that elicit a response by *D. longicaudata* includes acetaldehyde, ethanol, and acetic acid released by fungi that grow on decaying fruit [12], several compounds released by uninfested fruit [13] and acetophenone, a chemical originally identified from a floral volatile [14] but which may be “mistaken” for its analog, para-ethylacetophenone, a compound emitted by tephritid larvae and which elicits a response in sensilla on the ovipositor [15] (Figure 1).

Other opiines, such as *F. arisanus*, are also attracted to fruit volatiles [6,16], and *Pysttalia fletcheri* Silvestri, to decaying fruits and leaves of pumpkins and cucumbers (*Cucurbita* spp.; Cucurbitaceae) [17]. Earlier, Nishida [18] had found that stem tissues of cucurbits are attractive to *P. fletcheri*, and Messing, Klungness, Jang and Nishijima [17] suggested that the basis of the attraction was “green leaf volatiles”, a suite of common leaf-derived compounds known to be attractive to other braconid species [19].

The opiine *Utetes canniculatus* [Gahan] [= *Opius lectus*], as well as the pteromalid fruit fly parasitoid *Halticoptera rosae* Burks, are arrested by the Oviposition Deterring Pheromone [= ODP] of their *Rhagoletis* hosts and concentrate their searching on marked fruit. [20,21]. Other apparent arrestants are produced by uninfested fruit and can be used to stimulate oviposition in opiines such as *Doryctobracon areolatus* [Szépligeti] [22]. *Fopius ceratitivorous* Wharton is more likely to oviposit in eggs laid by a female fly than those artificially inserted into fruit [23], presumably because of cues left by the adult host such as an ODP or feces. Its congener, *F. arisanus,* is attracted to fruit fly feces and detects volatiles associated with tephritid eggs [24]. *Coptera occidentalis* Muesbeck, a Nearctic diapriid pupal parasitoid of *Rhagoletis* spp., follows water-insoluble trails left by host larvae as they wander and then burrow into the soil [25]. Previous attempts to document such trail-following by the related *Coptera haywardi* [Oglobin] failed, but *C. haywardi* is relatively competent at locating pupae buried at depths of <1 cm and is likely exploiting some chemical cue provided by the host [26,27].

#### 2.1.3. Vibration Cues

Hosts cloistered within their food are hidden from view, but they inevitably reveal themselves by vibrations made while moving or tearing at fruit pulp/seeds with their mouthhooks [3,28,29,30]. Of course, vibrations can travel both ways and those caused by probing parasitoids could elicit escape reactions by their host [31], although this remains to be determined in tephritids.

While feeding/movement circadian rhythms have not been discovered in tephritid larvae [32], other than a propensity in the laboratory for fully mature *Anastrepha suspensa* (Loew) larvae to remain in diet trays in the middle of the night rather than exit to pupate [33], an individual larva during molts or otherwise might spend some of its time motionless and undetectable by vibrations. For instance, certain individual stored grain pests can spend up to 29% of their time quiescent [34]. This could result in different parasitoid foraging niches and the exploitation of different host-cues, such as seen in parasitoids attacking *Drosophila* larvae, some of which appear to locate larvae through vibration and others by chemical cues [35,36]. Host size, *i.e*., age, could also affect the strength of larval vibrations [37] and so lead to parasitoids specializing on younger larvae to emphasize other cues.

#### 2.1.4. Plant Produced Signals

Are there any likely signals (evolved broadcasts of information) between plants burdened by tephritid infestation and natural enemies that could remove the pests? For example, a large number of plants from *Acacia* spp. to *Zea mays* produce volatile compounds when fed upon by herbivorous insects that attract parasitoids and predators [38]. In the case of frugivorous Tephritidae, there are two prerequisites for host-plant-broadcasters and tephritid-parasitoid-receivers to participate in a signaling system: (1) that tephritid larvae in fruit are costly to plant reproduction; and (2) that a natural enemy is capable of acting in time to prevent the damage (alternatively parasitoids might be exploiting chemicals released to attract immediately-valuable predators or even repellents directed toward the pest itself; e.g., [39].)

Those tephritid larvae that consume seeds, e.g., *Toxotrypana* spp., some *Rhagoletis* spp. and many *Anastrepha* spp., cause direct damage to a plant’s progeny and those that render the fruit pulp less attractive and nutritious to seed dispersers do so indirectly [40]. However, the death of the fruit fly may come too late to matter in the cases of koinobiont parasitoids that attack immature hosts but then eclose following their maturation (assuming parasitized hosts continue to feed in the same destructive manner; [38] or idiobionts that oviposit in pupae). From the perspective of a fruiting plant there is little advantage to investing in a signal that will bring help after the damage is done.

There are special cases among opiine fruit fly parasitoids where signals could be produced in synchrony with harm. Perhaps one of the more plausible is that those species that puncture host eggs, e.g., *Fopius* spp., cause many of the eggs in a clutch to die before hatching [41]. The death of a majority of immature tephritids prior to feeding might benefit a plant’s seed-offspring so that a signal that attracts egg-killing parasitoids would be worth the energy and materials to produce. However, the diversity and abundance of such parasitoids varies and they are uncommon or absent in many guilds. As far as is known, there is no co-evolved egg-attacking parasitoid of *Anastrepha* and in North America the only egg-prepupal tephritid-attacking braconids we are aware of are in the *Utetes caniculatus* complex that parasitize *Rhagoletis* spp.

#### 2.1.5. Host Vulnerability

**Figure 2:**
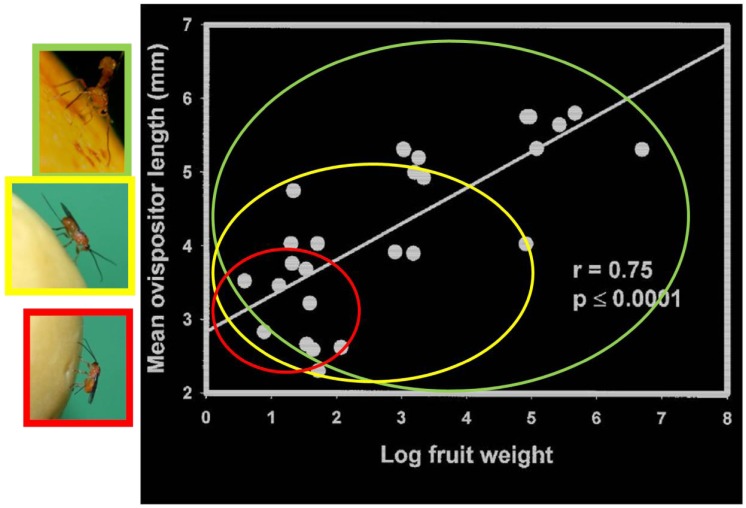
Ovipositor length influences how effectively a female opiine braconid can reach host larvae in various sized fruit, hence the positive relationship between the mean ovipositor lengths of parasitoids recovered from fruit and fruit weight. This is illustrated diagrammatically by the colored circles representing the ranges of fruit sizes accessible to host-foraging parasitoids with descending ovipositor lengths (green = *Diachasmimorpha longicaudata*, yellow = *Doryctobracon areolatus*, and red = *Utetes anastrephae*) [44].

Larvae sheltered deep within fruit pulp present a challenge to their parasitoids, and in the Opiinae the ovipositor is the tool they use to address the problem. While figitids and chalcidoids that attack tephritid larvae enter fruit through pre-existing or self-made ruptures in the skin and then crawl through the pulp to reach their hosts [42], braconids must “drill” from the surface and the length of the ovipositor limits the depths that can be plumbed [43,44,45,46]. Among the species within the Mexican guild of fruit fly parasitoids there are considerable differences in ovipositor length, both actual and relative to body length and as a result short-ovipositor species are mostly limited to a subset of smaller fruit and the mean ovipositor length of parasitoids emerging from fruit is strongly and positively correlated to fruit size [44] (Figure 2). In addition to size, fruit characteristics such as epicarp and pulp depth and firmness, and seed size and morphology are also likely to have a bearing on host accessibility [43].

Why ovipositors of parasitoids that attack the same stages of the same hosts in some of the same fruit should vary substantially in length is something of a puzzle. Perhaps longer ovipositors are more expensive to maintain and carry from site to site or more likely to buckle when attempting to penetrate harder fruit [47]. Of course a means of avoiding the problems of large fruit and unreachable host-larvae is to oviposit into shallowly deposited host-eggs. But tephritid eggs seem to be relatively fragile, resulting in high parasitoid mortality [41,48] and may be both ephemeral and provide fewer location-cues to foraging females, although often high parasitism rates seem to belie this possibility; see [23]. This could account for the typically greater proportion of larval parasitoids in tephritid parasitoid guilds.

Fruit sizes differ among species, among the trees of a particular species and even within the canopies of individual trees, and these size patterns can underlie the distributions of fruit flies and larval parasitoids. For example, in a study of five Mexican *Anastrepha* spp. in seven species of host trees attacked by five species of parasitoids, those parasitoids with longer ovipositors had broader host/host-plant ranges. Even in relatively small-fruited host-trees the parasitoid species with the shortest ovipositor was most abundant in interior portions of the canopies where fruit tended to be smaller still, and within the fruit of individual trees smaller fruit with higher larval densities contained a greater proportion of parasitized flies [49,50].

#### 2.1.6. Competition Among Parasitoids

A number of parasitoid species often exploit a particular host species [51]. This coexistence may be possible because of selection for resource portioning and if so, niche divergence can imply a cost to competition and a history of its avoidance. These escapes from competition can be accomplished through different foraging behaviours. For example, among the Neotropical opiine tephritid parasitoids only *Doryctobracon crawfordi* (Viereck) regularly forages on fruit fallen to the ground [52]. Species such as *D. areolatus* and *Opius hirtus* (Fischer) appear to be particularly adept at locating low density hosts and so might persist where other species are unable to maintain viable populations [52].

Another means of avoiding competition is through conditional-foraging. Female parasitoids that discriminate against already parasitized hosts, typically by sensing an ODP or faeces deposited by a previously ovipositing female, are said to avoid competition *indirectly*. Conspecific, even individual, ODP recognition is common and widespread in the Hymenoptera, and occurs in at least some fruit fly parasitoids [28,53]. However, recognition of heterospecific ODPs is relatively rare [54]. Even so, the opiine *Diachasmimorpha tryoni* (Cameron) discriminates against hosts previously parasitized by *F. arisanus*, [55]. The ratio of ovipositor-penetration scars to actual ovipositions in hosts already parasitized by *F. arisanus* is double that observed in unparasitized hosts. *Coptera haywardi*, a pupal endoparasitoid, avoids hosts previously attacked by *D. longicaudata* [56]. The latter is a superior *intrinsic* competitor, i.e., in cases of multiparasitism its larvae regularly suppress the immature stages of the former. Because the two species have only recently come to occupy the same region, the capacity of *C. haywardi* to avoid *D. longicaudata* likely evolved in yet another competitive context. Preliminary data suggests there are still other cases of recognition of prior heterospecific parasitism among tephritid parasitoids [57], and it is unclear why this otherwise rare phenomenon seems to be relatively common in this particular guild.

#### 2.1.7. Host Range Breadth

Some parasitoids develop in a single species of hosts, others in hundreds, and there is an enormous amount of literature that attempts to account for the evolution of host range breadth [58]. Aluja and Mangan [59] have formalized the terms used to describe host range breadth in fruit flies and these terms are equally useful in considering their parasitoids. Monophagous frugivorous-tephritid parasitoids are restricted under natural conditions to a single host species (perhaps *F. ceratitivorous*), stenophagous to a single host genus (*O. hirtus*), oligophagous to a single family (e.g., fruit-infesting tephritid-attacking opiines in general and *D. longicaudata* in particular; also the diapriid pupal parasitoid *C. hawardi*) and polyphagous to multiple families (e.g., chalcidoid idiobiont pupal parasitoids of Diptera; [60]).

Given the complexity of the topic we only note here that host-cues can be specific to a particular tephritid or common to many and that the responses to cues can be fixed or modified through experience [61,62]. Those parasitoids that attack a variety of fruit fly species must locate these hosts through cues produced by all in common or respond to a set of specific cues unique to each. The use of common cues is suggested by rapid adoption of novel hosts. For example, *D. longicaudata* has a wide host range within the Tephritidae, including species of *Anastrepha* it has only encountered within the last 70 years and on which it inflicted immediate and severe mortality [1]. As previously described it orients to general cues such as vibrations made by moving/feeding larvae, volatiles released by fungi associated with decomposing fruit and chemicals apparently released by all tephritid larvae [6,15,28].

There is evidence of reliance on more specific foraging cues, and these have been sometimes found in more specialized parasitoids. For example, *F. ceratitivorous* is an east African parasitoid of *Ceratitis capitata* (Wiedemann) brought to Guatemala where it has been released on a large scale against persistent pest populations in the coffee-growing highlands along the Mexican border [63]. It behaves differently when encountering an oviposition puncture made by other tephritid species and shows only marginal interest in the eggs of *Bactrocera* spp. and none in gall forming tephritids [61]. Even within parasitoid species with somewhat broader host ranges there may be specific host preferences mediated by specialized cues. The opiine *D. alloem* (Muesebeck) attacks both *Rhagoletis pomonella* (Walsh) in hawthorn and *R. mendax* Curran in blueberry, but prefers the odor of the fruit that its larval-host developed within [62].

#### 2.1.8. Abiotic Influences on Foraging

Tephritid parasitoids differ in their regional distributions and differences in temperature and humidity have long been thought to contribute to the limits of individual species distributions. Low winter temperatures were believed to prevent the establishment of *F. arisanus* in southern Australia [64,65] and Darby and Knapp [66] noted that the opiine *D. crawfordi* was less tolerant of low temperature than its host *Anastrepha ludens* (Loew). More recently, Wang, Levy, Son, Johnson and Daane [67] found that *Psyttalia lounsburi* (Silvestri) was less heat tolerant than *Bactrocera oleae* (Rossi), while *Psyttalia humilis* (Silvestri) was more so, and that this is likely the result of adaptations to local environments within the historically expanded range of the fly. In Florida, the introduced species *D. longicaudata* and *D. areolatus* have disjunct distributions, southern and northern peninsular respectively, with very little overlap [68]. Temperature, as well as competition, may contribute to this difference since the northern limit of *D. longicaudata’*s range is remarkably close to the mean minimum winter isotherm for the state and its abundance is negatively correlated to the variance in monthly temperatures. One model to explain the relationship between distributions and temperature variance posits that although both species can enter diapause [69], *D. areolatus* is better able to bridge gaps in host availability and thus persists in more temperate regions were host-fruit diversity is lower and time between seasonal oviposition opportunities is longer.

Altitudinal distributions, like those based on latitude, are assumed to be related to changes in temperature/moisture and their effects on either parasitoids or their hosts/host plants. In southern Mexico, parasitoid guild diversity and composition, as well as parasitism rates, change substantially along transects running from sea level to 2000 m [70].

Beside regional temperature differences in environment, tephritid parasitoids ensconced within hosts occupying different parts of tree canopies are subjected to significant differences in temperature which in theory could influence developmental time and survival [71]. In Florida, fruit hanging in the southwest portions of tree canopies are warmer than those in the northeast and fallen fruit on the ground can be up to 15 °C warmer than fruit hanging in the canopy above. This range of microhabitats may underlie fly distributions within canopies either because of foraging-adult tolerances or preferences based on larval performance. For example, *Anastrepha striata* Schiner tends to be more abundant in the upper portions of its myrtaceous host trees, while *Anastrepha obliqua* (Macquart) density is greater in the lower of its anacardiaceous host trees [72]. In general, rates of parasitism are higher in the lower portions of certain host-trees, but differences in fruit sizes and competitive interactions are also likely to have a significant effect on these distributions (49,50).

#### 2.1.9. Foraging for Hosts and Augmentative Biological Control

**Figure 3:**
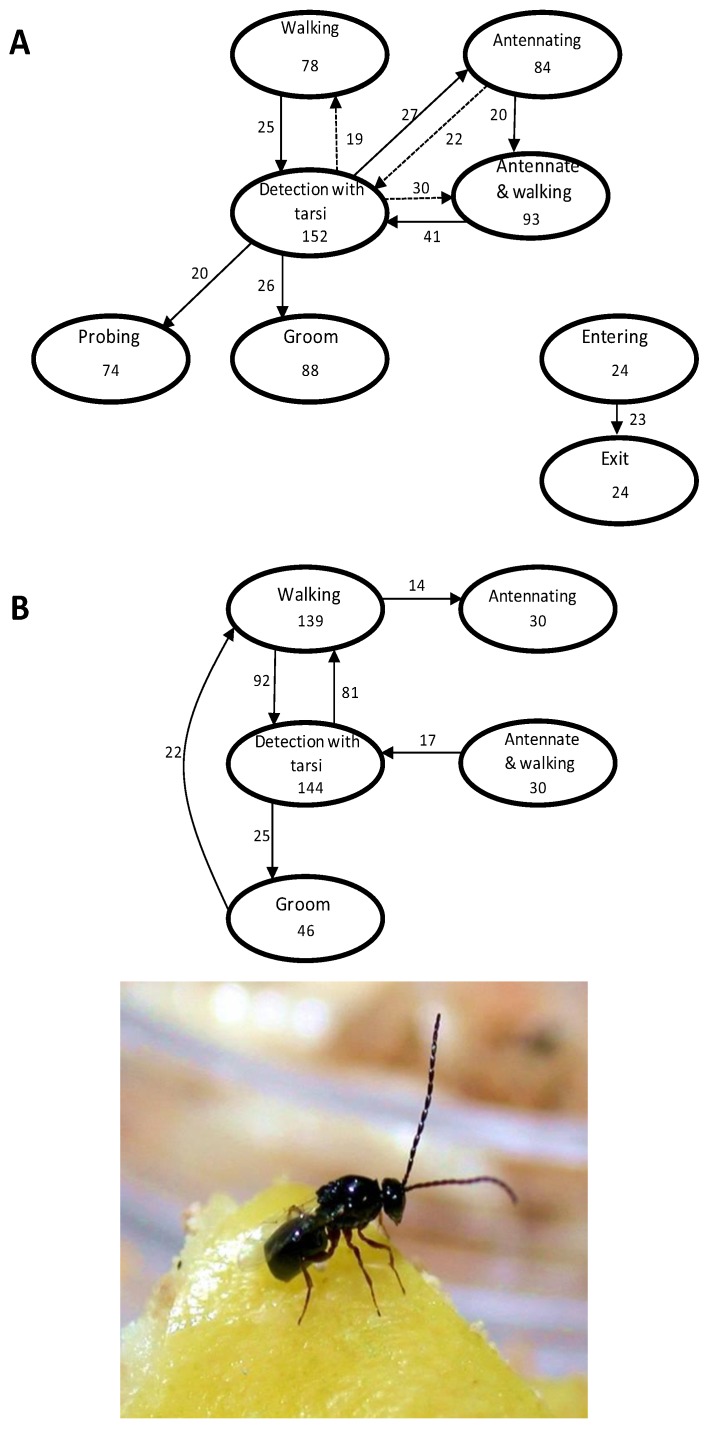
Because a mass-reared tephritid parasitoid is inevitably more expensive to produce than an adult fruit fly, cost saving measures will increase the feasibility of augmentative releases relative to the Sterile Insect Technique. All female strains, such as those based on *Wolbachia-*induced thelytoky, could cut rearing expenses in half. However, there may be behavioral and physiological consequences to *Wolbachia* infection. Thelytokous *Odontosema anastrephae* (Figitidae; inset) (**b**) have simplified host-searching repertoires compared to arrhenotokous females (**a**). The consequences of this simplification to biological control are unknown. Ovals represent behavioral acts, numbers inside the boxes are behavioral repetitions. Solid arrows represent transitions significantly different from expected (non-random), and dashed arrows represent transitions not significantly different from expected (random) [83]).

All of the above, the requirements of parasitoids and their hosts, the biotic and abiotic components of the target environment, how the insect’s needs and limitations are perceived and responded to, contribute to the planning, execution and success of an augmentative parasitoid release program. Some particular points to consider and research proposed would include:

(1) Argumentatively released parasitoids capable of locating hosts in low-density populations might continue to inflict mortality on a target population after other species disperse from “poor-quality” habitats. For this reason, relaxed density-dependent foraging, Force [73] argued that biological control practitioners should closely consider the marginal portions of a pest’s original range and pay particular attention to the parasitoids that manage to persist there. With this in mind and all other things equal, *O. hirtus* might make an attractive candidate for the augmentative control of *Anastrepha* spp. [52]. Those interested in exploring the question of density-dependent parasitoid foraging, and ultimately its potentially synergistic interaction with the Sterile Insect Technique, would do well to first read Heimpel and Casas’s review [74].

(2) Microhabitats can influence the opportunities available to a parasitoid, e.g., *D. longicaudata* could continue to forage over late-season fallen and rotting fruit [75] that would fail to attract the attention of many native Neotropical opiines. In addition to questions of release timing and foraging efficacy such microhabitat preferences can complicate the estimation of parasitism rates and project efficacy. Parasitism inflicted by species that forage largely within tree canopies can be accurately determined by sampling and holding recently fallen fruit. This procedure would underestimate the effect of species that continue to oviposit into late-instar larvae in fruit as it rots upon the ground, i.e., smaller larvae in the fruit would not be vulnerable to parasitoids and if allowed to mature following collection and then counted would inflate the numbers of tephritids that had escaped attack [52]. Sampling schedules, such as only counting the first one-three days of larval emergence from sampled fruit (reflecting only larvae that could have been at risk to certain parasitoids), somewhat mitigate parasitism underestimation [76], but only complete exposure of fruit and flies in the field will yield accurate results [75].

(3) Hymenopteran parasitoids can associatively learn host- and food-finding cues [77] and this suggests that post-release movement towards particular microhabitats and host choice specificity could be manipulated by pre-release experiences. *Fopius arisanus* that learned to identify a substrate containing hosts, laid more eggs and produced more offspring than those without prior experience [78]. In both the field and the laboratory, adult fruit fly parasitoids preferred their developmental-host’s substrate [62,79]. The ability to focus parasitoid on more profitable or accessible hosts could influence the immediate efficacy of released parasitoids and deserves further investigation.

(4) From a control program perspective, a mass-reared parasitoid should be able to prove itself more valuable than its alternative, generally several sterile male fruit flies, given the expense of rearing. One means of making augmentative biological control’s cost: benefit ratio more attractive is to develop/discover thelytokous parasitoid strains consisting of *Wolbachia* infected females. All female offspring should cut rearing costs roughly in half [80], but this presumed advantage supposes parasitoid value, i.e., foraging capacity and other life-history characteristics, is unaffected by cellular endosymbionts. This is not always the case. For example, in some *Wolbachia-*infected *Trichogramma,* a pronounced loss of fecundity is due to failed mitotic divisions in embryos [81]. Comparisons of arrhenotokous and *Wolbachia-*infected lines of *Trichogramma* spp. found that the arrhenotokous insects had higher fecundity and dispersed further in laboratory tests [82]. However, there were no differences in dispersal in greenhouses, and the absence of males in the thelytokous lines resulted in greater biological control when identical numbers of the two lines were released. Thelytokous *Wolbachia-*infected females of the figitid fruit fly parasitoid *Odontosema anastrephae* Borgmeier have simplified foraging behaviors relative to insects from uninfected populations, although the consequences of fewer behavioral steps in the parasitization process is unknown [83] (Figure 3).

Infected females of the same species live a shorter time under crowded laboratory colonies, similar to conditions in mass-rearing [84] which makes population growth rates actually lower than those of bi-sexual colonies. However, evidence is accumulating that *Wolbachia* protects its hosts from other pathogens so that the survival of released infected-parasitoids in the field may prove to be enhanced [85].

(5) Host larvae hidden deep in fruit pulp/seeds present challenges to parasitoids. It is often beneficial to target fruit fly larvae in small, often native or otherwise non-commercial fruit, as opposed to domesticated, artificially enlarged commercial fruit [44,46]. Not only are larvae more vulnerable in such fruits but they often make up the refugia from which flies spread to threaten commercial plantings. Typically there is little profit in parasitizing larvae that have already infested the crop and so ruined its value [76].

(6) Different stages of host development have different vulnerabilities and all things being equal eggs are more shallowly placed than larvae and so more exposed to parasitoids [41]. If only larval parasitoids are available then ovipositor length and it relationship to parasitism with increasing fruit size should be taken in account. There are a number of fruit-entering figitids and eulophids that in theory could reach larvae regardless of fruit size. In practice they typically inflict low parasitism levels [45,70]. Mature larvae leave the shelter of their fruit to pupate, but then are hidden underground. In the laboratory, some pupal parasitoids such as *Pachycrepoideus vindemmiae* Rondani [Pteromalidae] have little success reaching even shallowly buried hosts [27], but others like *C. haywardi* can inflict over 40% mortalities on pupae buried at ~5mm under semi-natural conditions [26].

(7) Since not all tephritid pests are attacked by all parasitoids the appropriate choice of natural enemy is paramount. It would be futile for example, to release any of the native Neotropical opiines to attack *C. capitata* or expect *F. ceratitivorous* to efficiently parasitize Neotropical *Anastrepha* spp.

(8) Not only is it important that a parasitoid attacks the target pest but also that it does not attack non-targets which can include rare non-pestiferous congeners or even seed-head attacking tephritids that were themselves introduced for the biological control of weeds. This can be a challenge since the natural history and even the hosts of many Tephritidae are unknown and parasitoid host preferences can change rapidly when confronted with new environments and competitors [86,87]. Another form of “non-target” at risk is the pre-existing and locally-adapted populations of the parasitoid being mass-reared and released. The gene pool of such populations could be diluted by a flood of wasps from another location or that have lost through domestication genes beneficial in the field [88] (see however [77]).

There is a considerable range of host breadth within the opiine koinobiont endoparasitoids of fruit flies and this should be taken into account as the first step in avoiding unnecessary risks. There are sometimes a number of candidates and even among pupal parasitoids, typically idiobiont ectoparasitoids capable of consuming entire suborders of Diptera, there are relatively oligophagous species such as the tephritid-restricted and endoparasitic *C. haywardi* [89,90]. Some parasitoids develop in a particular pest, but do not subsequently become established probably because of inhospitable environments or seasonal gaps in host availability, e.g., *F. arisanus* throughout the neotropics [1]. From an environmentalist perspective these are particularly attractive candidates since they disappear following augmentation and thus do not pose a “permanent” threat to non-targets.

In the past the economic benefits of introducing opiines were assumed almost without question to outweigh any environmental risks, but this is no longer the case and Messing and colleagues [87] have been at the forefront of developing experimental approaches and techniques to quantify the environmental impacts of fruit fly parasitoids.

(9) Severe interference competition can occur among tephritid parasitoids, as illustrated by the rapid change of guild composition when parasitoid species are sequentially introduced into novel situations. Classic cases are opiine braconid parasitoids of exotic tephritid fruit flies in Hawaii and Florida, where flourishing natural enemies were quickly replaced over large portions of their ranges by new introductions [66,91]. Interspecific-competition poses an age-old question among biological control practitioners: whether it is better to attempt establishment of a single or multiple species. That is, would a parasitoid inflict the same mortality and have the same host range in the absence of competitors. Perhaps this is not as important a question from the stand point of augmentative releases unless establishment could be an unintended consequence. As previously noted host range shifts among tephritid parasitoids have occurred following the introduction of a superior competitor [86]. Conservation of natural enemies through landscape manipulation avoids some of the issues associated with introductions. A scheme has been proposed to plant native trees, attacked by non-pestiferous flies but sharing parasitoids with pest species, in agricultural settings [92]. Such “banker plants” would act as natural enemy reservoirs, perpetuate threatened plants and flies and in some cases serve as sources of valuable timber. Both *D. longicaudata* and *Utetes* sp. are attracted to certain flowers and their addition to agricultural landscapes might concentrate parasitoids [14,93].

(10) Tephritid parasitoids can have marked differences in their altitudinal and latitudinal distributions and these are likely to be in part the result of different preferences for temperature and humidity [68,94]. Obviously, the choice of parasitoid for mass-rearing and release should take the target area-environment into account.

(11) Loading insects into aerial mass-release devices and delivering those insects to their target typically requires up to an hour of chilling at temperatures of ~3.5 °C. Chilling three *Diachasmimorpha*, spp. and *F. arisanus*, all species established in warm tropical environments worldwide, to that level and for that amount of time had little or no effect on their longevity, fecundity or offspring sex-ratio [95]. A fall from an aircraft *per se* did not affect either the longevity of *D. tyroni* or its capacity to take flight [96].

(12) Efficient and cost-minimizing rearing procedures should incorporate optimal development temperatures, many of which need to be determined [97].

### 2.2. Foraging for Food

**Figure 4:**
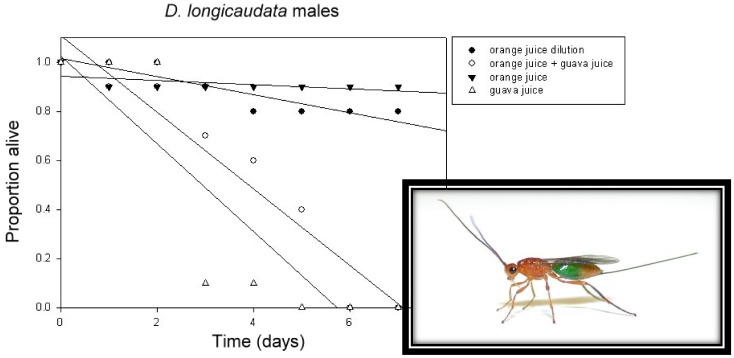
Obtaining adult food from the same substrate occupied by hosts saves the energy and avoids the risks inherent in separate foraging. Although opiine braconids flourish on various fruit/ fruit juices, such as orange (*Citrus sinensis* L.) and peach (*Prunus persica* [L.] Batsch), others appear to be toxic. For example, parasitoids fed on guava (inset of *D. longicaudata* that had consumed marked juice) die quickly, even when guava is diluted with nutritious orange juice. As a consequence not all environments may offer the same opportunities to safely obtain cheaply-acquired carbohydrates. Thus different habitats may require different food-foraging tactics. Volatile cues are used by tephritid-attacking braconids to locate fruit juices and as might be predicted, those from a high quality food such as orange are generally more attractive than those of low quality guava. [6,100].

Adult opiine parasitoids require carbohydrates [98] and as a rule honey or honey and water have been provided to feed mass-reared parasitoids (e.g., [99]). Sugar solutions, fructose or sucrose, have also been successfully employed [100]. The sources of necessary carbohydrates in nature are not well identified. There is little, and that ambiguous, evidence of floral-feeding in *D. longicaudata,* but it does well on hemipteran honeydew, extra-floral nectars and particularly the juices of certain fruit that could be made available from mechanical ruptures in fallen fruit or oviposition wounds [101]. Since parasitoids incur additional costs and risks foraging separately for both food and hosts [102], obtaining both in the same fruit-microenvironment could have substantial advantages. However, fruit juices vary in nutritional quality and guava, *Psidium guajava* L., is toxic to several opiine species [100,103] (Figure 4).

Opiine parasitoids without carbohydrates die within 48–72 h [101]. Releases into regions where adult food is unavailable are doomed either because of almost immediate parasitoid mortality or dispersal. Under many circumstances infested fruit, the target of the control enterprise, could also serve as a source of nutrients, but there might be some peculiar situations, like extensive plantings of toxic guava without honeydew producing Hemiptera, where sugar-spray supplements might be considered [98,103]. Both a pteromalid and an opiine parasitoid of *B. oleae* prefer honeydew to a spinosad GF-120 bait and do as well on honeydew as honey [104].

#### 2.2.1. Foraging for Food and Augmentative Biological Control

(1) A synthetic attractant is needed to monitor the survival and dispersal of released parasitoids. There are at least six potential sources of attractive compounds, in addition to adult foods, fruit, honeydew or flower-nectar [14,98,101,103]. There are sex pheromones, fruit fly larvae [15], the by-products of fruit decay [12], infested fruit volatiles such as 2-phenylethyl acetate unique mango (*Mangifera indica* L.) [105] and adult-host semiochemicals [20,21]. However, volatiles released from foods as opposed to those associated with oviposition substrates might be attractive to both males and females and so have higher capture rates. Male-produced sex pheromones are often ignored by mated females and so are generally not as attractive as female-produced. There is evidence for both types in opiines but sexual communication is presently poorly understood (see below). Optimally attractive trap colors and shapes, if important, need to be determined for most candidates for augmentative release.

### 2.3. Foraging for Mates

#### 2.3.1. Mating Systems

Surprisingly little is known about tephritid-attacking opiine mating in nature. A detailed study was performed in Australia of the introduced *F. arisanus* [106] and there have been brief observations of two species, *D. longicaudata* and *D. areolatus*, occurring in Florida [107]. All these mating systems involved aggregated males which seemingly emit a pheromone and produce a wing-fanning acoustic signal from a defended leaf-territory on or under a host-fruit tree [108]. Chemical and acoustic “calling” on the territory alternated with swoops to the ground. These two activities may represent two sexual tactics, to search for females as they emerge from buried fly-puparia and then signal to receptive females that were not mated by a competitor as they left the soil [106,108]. *Fopius arisanus*’ mating system has additional complexities. Unlike the majority of braconids (including *D. longicaudata* [109]), males are sexually immature for several days following eclosion [110] and this seems to have led to an age-based stratification of males in the foliage in and around host trees. Older, sexually active males perch on grasses and foliage underneath host tree canopies and young, sexually immature males are in the host trees themselves. Thus, males ready to mate are closest to emerging females while immature males remain in the general vicinity of emerging females but avoid any male-male competitive interactions that might be occurring closer to the ground.

#### 2.3.2. Sex Pheromones

Male opiine fruit fly parasitoids produce distinctive, often “pleasant”, odors associated with compounds derived from Hagen’s gland located on the abdominal tip; *F. arisanus* has a coconut-like aroma [110,111]. Given their pre-copulatory sexual behavior, which can include abdominal taps leaving moist marks upon the substrate, exposure of membranes on lofted abdomens and wing-fanning (often associated with pheromone dispersal; see below), there is a widely held assumption that males produce a chemical sexual signal [111]. However, this has not been a universally held opinion. Buckingham and Sharkey [112] believed the gland’s products, at least in part, to be defensive secretions, and there is negative experimental evidence of female attraction to chemical signals in the field, *i.e*., female *F. arisanus* were not captured in sticky traps baited with live males [106]. That said, the chemical diversity of the gland’s contents suggests a communicative function rather than a evolutionarily conserved means of defense [111]. Preliminary experiments using a variety of laboratory bioassay devices found that male*D. longicaudata* attract both virgin females and other males [113]. Females in the field have been seen directly approaching male aggregations from a distance and the absence of females in male-baited traps could be because unmated females are relatively rare in nature and mated females are unlikely to respond to males’ signals. On the other hand, female *F. arisanus* do attract males into sticky traps [106] and male *D.alloeus* respond to crushed females but not *vice versa*[114]. Male *P. concolor* court and attempt to copulate with both living and recently killed females, but not if the dead females have had their chemical contents partially removed by soaking in hexane [115].

#### 2.3.3. Acoustic Signals

Repeated wing-generated buzzing sounds produced by male opiines, particularly in proximity to conspecifics, act as close-range courtship, and perhaps agonistic, signals (= songs) [116]. Such songs have been proposed to have originated from wing-fanning used to disperse pheromones that secondarily came to serve as a measure of male vigor (an example of Marshall McLuhan’s famous dictum that …“the medium is the message”).

#### 2.3.4. Foraging for Mates and Augmentative Biological Control

(1) Our experience has been that mortality-by-parasitoids in control programs is often discussed as a means of immediate population suppression, particularly if parasitoid releases are repeated at intervals of one or two weeks or quickly followed by the application of sterile male flies. However, this does not mean that successful mating, and the female offspring that result, could not be significant addition to the pool of parasitoids active in the field [117]. Experiments with increasing lengths of time between releases might reveal the importance of the “F1” progeny of released females and perhaps lead to cost-saving modifications of release schedules.

(2) *Anastrepha suspensa* suppression by augmented *D. longicaudata* in Florida was much improved when adults of both sexes were held together for several days prior to release [76]. While the cause of this improvement remains unknown it was believed at the time that both the provision of a high quality diet and an opportunity for females to mate in captivity were responsible [98]. The logic behind the latter was that females that did not have to search out mates were immediately focused on oviposition. This remains an area for further research.

(3) Sexual behaviors have proved to be useful indices of general quality in mass-reared tephritids [118] and perhaps the same could be true for their parasitoids. For instance, if acoustic signals could be correlated to general vigor or reflect the degree of genetic change under domestication then their measure might serve as a means of quality control in mass-rearing facilities. Joyce, Aluja, Sivinski, Vinson, Ramirez-Romero, Bernal, and Guillen [119] analyzed the songs of four Mexican-native and one introduced species from recently established colonies and those maintained for 70–148 generations. In four of five species there were differences, but at this time the significance of those differences, e.g., whether they are correlated to other qualities and if so whether the trends indicate greater or lesser vigor, is unknown. Male qualities preferred by females during mate-choice could parallel those sought by production managers. Larger males were more sexually successful in *D. longicaudata* and *P. incisi* but not in *Biosteres vandenboschi* (Fullaway) or *P. fletcheri* [109]. The songs of sexually successful male *P. concolor* had longer pulse duration and pulse intervals than those of unsuccessful males [120]. There remains a great range of research to be done in acoustic as well as in other aspects of opiine mating.

## 3. Conclusions

Reviews typically reveal more ignorance than knowledge and this one is no exception. There are enormous technical gaps in the practice of fruit fly augmentative biological control and these need to be filled before the efficacy of releases rise and their costs decline. Addressing many of these shortcomings will require more detailed descriptions of foraging behaviors. As an illustrative example let us ask a single important question and then follow the cascade of unanswerable complications that arise. How many parasitoids should be released to suppress a population of a particular tephritid in a certain environment? Simply measuring how long released parasitoids survive and where they go is difficult. Then it must be determined if their dispersal and parasitism rates are density dependent; *i.e*., as fly numbers fall will augmentation become increasingly ineffectual? Are density relationships the same for all the available candidates? Is there a speedy means of obtaining some of the information as opposed to fruit collections and parasitism calculations that may provide data long after its programmatic usefulness has passed? Is parasitism even the critical data to be gathered? In some situations, unsuccessful parasitism attempts by egg-prepupal parasitoids result in levels of host destruction that seem more important to pest suppression than the actual development of parasitoids. Could timely data on the fate of released parasitoids be obtained with traps? What sort of attractants would be deployed? It is striking how little is known about intersexual chemical communication and chemically-mediated tritrophic interactions in even the most widely reared parasitoids. If parasitoids are dispersing from release sites is this the result of depressed fly populations or exhaustion of adult food? What are the adult foods? We could continue, but this much suffices to make obvious that investigations into the foraging behaviors of tephritid parasitoids are important and that they offer abundant opportunities for research.
